# Wild and Domestic Differences in Plant Development and Responses to Water Deficit in *Cicer*

**DOI:** 10.3389/fgene.2020.607819

**Published:** 2020-12-04

**Authors:** Jens Berger, Raju Pushpavalli, Christiane Ludwig, Sylvia Parsons, Fatma Basdemir, Kelly Whisson

**Affiliations:** ^1^Commonwealth Scientific and Industrial Research Organization (CSIRO) Agriculture and Food, Wembley, WA, Australia; ^2^Ceylanpinar Agriculture Vocational School, University of Harran, Sanliurfa, Turkey

**Keywords:** crop wild relative, partitioning, water-deficit, water-use, domestication

## Abstract

There is growing interest in widening the genetic diversity of domestic crops using wild relatives to break linkage drag and/or introduce new adaptive traits, particularly in narrow crops such as chickpea. To this end, it is important to understand wild and domestic adaptive differences to develop greater insight into how wild traits can be exploited for crop improvement. Here, we study wild and domestic *Cicer* development and water-use over the lifecycle, measuring responses to reproductive water deficit, a key Mediterranean selection pressure, using mini-lysimeters (33 L round pots) in common gardens under contrasting water regimes. Wild and domestic *Cicer* were consistently separated by later phenology, greater water extraction and lower water use efficiency (WUE) and harvest index in the former, and much greater yield-responsiveness in the latter. Throughout the lifecycle, there was greater vegetative investment in wild, and greater reproductive investment in domestic *Cicer*, reflected in root and harvest indices, rates of leaf area, and pod growth. Domestic WUE was consistently greater than wild, suggesting differences in water-use regulation and partitioning. Large wild-domestic differences revealed in this study are indicative of evolution under contrasting selection pressures. *Cicer* domestication has selected for early phenology, greater early vigor, and reproductive efficiency, attributes well-suited to a time-delimited production system, where the crop is protected from grazing, disease, and competition, circumstances that do not pertain in the wild. Wild *Cicer* attributes are more competitive: higher peak rates of leaf area growth, greater *ad libitum* water-use, and extraction under terminal drought associated with greater vegetative dry matter allocation, leading to a lower reproductive capacity and efficiency than in domestic chickpea. These traits strengthen competitive capacity throughout the growing season and are likely to facilitate recovery from grazing, two significant selection pressures faced by wild, rather than domesticated *Cicer*. While increased water extraction may be useful for improving chickpea drought tolerance, this trait must be evaluated independently of the other associated wild traits. To this end, the wild-domestic populations have been developed.

## Introduction

Given genetic bottlenecks in most crop species, there is widespread interest in exploiting crop wild relatives (CWRs) that typically harbor much greater diversity (Tanksley and McCouch, 1997; Dempewolf et al., 2014; Gepts, 2014). Advocates of base broadening suggest that the greater genetic diversity of CWR will improve complex traits like yield by breaking linkage drag through the introgression of novel, previously unexploited alleles (Tanksley and McCouch, 1997), and/or be reflected in greater adaptive diversity (Dempewolf et al., 2017; Coyne et al., 2020). Indeed, ideas such as exploiting the climatic resilience of wild populations have gained considerable traction recently (McCouch et al., 2013; Dempewolf et al., 2014). Supporting this, there is an ever-increasing understanding of plant domestication in terms of history, geography, trait selection over time, and the genomic implications of these (Purugganan and Fuller, 2009; Meyer et al., 2012; Meyer and Purugganan, 2013; Abbo et al., 2014; Gepts, 2014; Larson et al., 2014). However, this work largely focuses on changes in the domesticate, rather than the attributes of the wild progenitor. As a result, when we want to turn to the wild relatives for crop improvement, we lack a framework to guide us. Genomic approaches maximizing genetic diversity to break up linkage drag advocated by Tanksley and McCouch (1997) are being deployed in breeding and pre-breeding programs in diverse ways from nested association mapping (von Wettberg et al., 2018) to MAGIC populations through to genomic selection (Bazakos et al., 2017). While these offer a methodology for increasing the genetic diversity of our breeding programs, they do not guide us in the identification of new adaptive traits and strategies that are missing in the cultigen, and which may or may not be present in the CWR (see discussion in Dempewolf et al., 2017). Thus, the big picture questions such as “where among wild populations do we find the adaptive traits with which to improve our crops and why?” remain important priorities (Thormann et al., 2014).

Clearly domestication has changed the adaptive capacity of crop compared to wild progenitor. In addition to the selection of domestication syndrome traits (lower seed dispersal and dormancy, larger seed size and more erect habit, and modified phenology), humans have altered many aspects of the cropping environment compared to that in which the progenitors evolved (Milla et al., 2015). Domesticated crops grow in managed (mostly) fields under reduced competition relative to the wild state (Gepts, 2014), their lifecycle is regulated (Milla et al., 2015), and they are not typically found as weedy escapes in the habitats, where their wild relatives occur. *Ipso facto* wild and domestic adaptive capacities do differ. But this does not mean that wild relatives will automatically contain all the adaptive traits lacking in the domesticate. Both wild and domestic plants are shaped by evolutionary responses to selection pressure which should be taken into account when evaluating their adaptive potential (Milla et al., 2015). There is a disconnect here between the agricultural and ecological approaches to this problem. While the former often samples widely (see examples in Singh et al., 1990, 1995, 1998), when screening for a shopping list of required traits ignores questions such as how representative is our collection and what selection pressures were imposed by the environment of origin, no greater insights into the biology of the species are made. While ecological approaches do address these issues, their experimental material may lack the depth to provide certainty. Recently, an argument has been advanced that under domestication crops evolved into resource-acquisitive, fast growing plants (competitors *sensu* Grime, 2006) as a result of their cultivation in well-managed, resource rich environments relative to those of their wild progenitors (Milla et al., 2015). By extension, wild progenitors should be relatively slower growing plants with traits tending toward the stress tolerator spectrum *sensu* Grime (2006). This is an interesting idea which has been tested quite widely across species, but with little depth (typically one wild and domesticated accession per species), and only in the early vegetative phase (Milla et al., 2014; Matesanz and Milla, 2018), where the strong selection for early vigor in domesticated crops (Evans and Dunstone, 1970; Berger et al., 2017) might be expected to influence the results.

These concepts are particularly tractable in wild and domestic *Cicer*. The genetic narrow base of chickpea as an adaptive constraint has been long recognized (Abbo et al., 2003). Early work at the International Center for Agricultural Research in the Dry Areas (ICARDA) demonstrated that the wild *Cicer* had a wider, potentially useful range of responses to pests, diseases, and stresses than domestic chickpea, particularly for ascochyta blight, leaf miner, bruchids, cyst nematode, and vegetative cold (Singh et al., 1998). However, at that time the world collection of wild, *Cicer* was far too narrow to adequately characterize the adaptive potential of any species, including those that can readily cross with domestic chickpea, with only 18 independent accessions of *Cicer reticulatum*, the wild progenitor, and even less for its close relative, *Cicer echinospermum* (Berger et al., 2003). This has changed recently with extensive new collection across the habitat range of both these species in Southeastern Anatolia (von Wettberg et al., 2018) that is driving renewed interest in trait discovery in these CWRs (Reen et al., 2019; Newman et al., 2020).

In this paper, we investigate wild and domestic *Cicer* responses to reproductive water deficit (terminal drought), one of the principal selection pressures exerted by the Mediterranean climate, using mini-lysimeters in common gardens under contrasting reproductive water regimes. This approach allows us to compare wild and domestic responses to contrasting resource (water) availability as well as the underlying water-use patterns. This is followed up by a plant above‐ and below-ground development and water-use study to describe wild-domestic differences in greater depth across the lifecycle. We were interested to discover to what extent the wild and domestic *Cicer* would segregate along stress tolerator-competitor continuum of Grime (2006). Secondly, we were interested to explain a consistent field observation made during the collection that the annual wild *Cicer* species tend to have a longer lifecycle, reproducing and maturing considerably later than most of their sympatric annual plant competitors such as *Lens*, *Pisum*, and many *Vicia* species. To validate the evolution of “acquisitiveness” in domestication hypothesis, the following expectations should be met:

1. Lower growth rates, water-use, and above‐ and below-ground productivity in wild compared to domestic.
2. Improved stress tolerance in wild compared to domestic.
3. Lower response to resource (water) availability in wild compared to domestic.

## Materials and Methods

### Rainout Shelter Water Deficit Studies-Overview (Exp 1–4)

**Figure 1:**
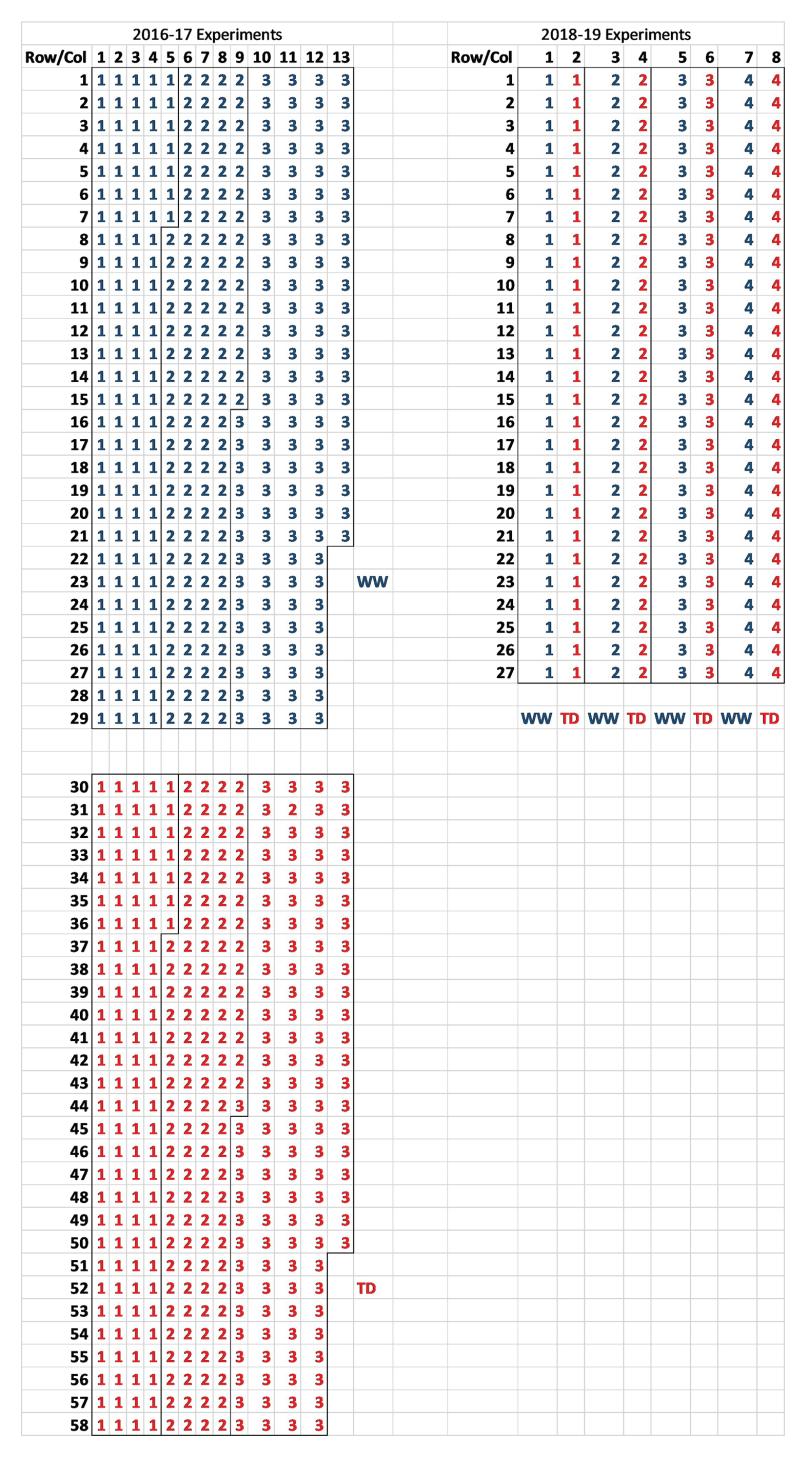
Plot layout of the 2016–2017 randomized complete block design (RCBD; *n* = 3) and 2018–2019 split-plot (*n* = 4) rainout shelter trials at CSIRO Floreat listing block identity for each plot [red for terminal drought (TD) and blue for well-watered (WW)].

A series of reproductive phase water deficit studies were run in the Commonwealth Scientific and Industrial Research Organization (CSIRO) Floreat rainout shelter from 2016 to 2019 comparing wild and domestic *Cicer* species collected from contrasting environments (Table 1). CSIRO Floreat is located in Perth, Western Australia (31.95°S, 115.79°E), a Mediterranean-type climate. The experiments were conducted in the standard Mediterranean late autumn to late spring growing season. The two larger 2016–2017 experiments (see Table 1 for accession details) were randomized complete block designs (RCBDs, *n* = 3) randomized within water regime [terminal drought (TD) and well-watered (WW)]. Because of space limitations, the two water regime treatments were allocated individually in two contiguous areas along the rainout shelter bay separated by 2 m (see Figure 1 for layout). This was done so that the rainout roof would shelter only the TD treatment when rain was detected. Rainout shelter closure was an automatic sensor driven process whereby the roof covers the TD treatment during rainfall and then withdraws. The 2018–2019 experiments were smaller split plot designs (*n* = 4) with water regime as main plots, accessions as sub-plots, and all located in the same parcel of the rainout shelter bay (see Figure 1 for layout).

**Table 1:** Accession numbers evaluated for water deficit response in the Commonwealth Scientific and Industrial Research Organization (CSIRO) rainout shelter from 2016 to 2019, categorized by species and collection site (accession counts within species presented in bold type).

Sp/collection site	Dry-down 2016	Dry-down 2017	Dry-down 2018	Dry-down 2019
***Cicer arietinum***	**3**	**3**	**4**	**4**
Domestic (Aust desi)	1	1	1	1
Domestic (Aust kabuli)	1	1		
Domestic (Indian desi)	1	1	3	3
***Cicer echinospermum***	**30**	**39**	**11**	**11**
Cermik	3	5	1	1
Destek	5	11	1	1
Gunasan	2	1	1	1
Karabahce	8	11	2	2
Ortanca	2	1	1	1
Siv-Diyar	10	10	5	5
***Cicer reticulatum***	**102**	**82**	**12**	**12**
Baristepe 1	8	8	1	1
Baristepe 2	5	3		
Baristepe 3	8	6	1	1
Beslever	7	6	2	2
Cudi	9	4	1	1
Cudi 2	9	4	2	2
Dereici	10	7	1	1
Egil	6	4	2	2
Kalkan	6	3	1	1
Kayatepe	7	6		
Kesentas	9	7		
Oyali	6	7	1	1
Sarikaya	9	2		
Savur	1	1		
Sirnak	2	14		
**Grand total**	**135**	**124**	**27**	**27**

Bold values are species summaries and grand total.

Experimental plots were 33 L round pots (430 × 340 mm) containing ca. 38 kg of Gingin loam. Five evenly spaced plants were planted in each pot (one in the middle and one in each quadrant).

### Germplasm

The 2016 and 2017 trials evaluated a wide range of wild germplasm (*C. echinospermum* and *C. reticulatum*) collected from a range of sites against domestic chickpea check varieties (Table 1). The 2018 and 2019 experiments evaluated a subset (*n* = 27) of this material, choosing contrasting accessions based on the previous results.

### Water Regimes

Reproductive water deficits were set-up by with-holding water in the terminal drought (TD) treatment when pod set was underway, defined by the first appearance of enlarged, but unfilled pods. To this end, phenological observations (dates of first flowering, podding, and pod enlargement) were recorded three times a week. The WW treatment was irrigated three times weekly until the end of the experiment. Typically, the TD dry-down phase took 16–23 days to complete, at which point the WW treatment was also stopped. During the vegetative phase, all plants were largely rain-fed, and only manually watered occasionally when required. Vegetative phases across years were consistently wet and cool (Table 2).

**Table 2:** Mean temperatures and rates of change for dry-down (DD) groups evaluated in the 2016–2019 water deficit trials at CSIRO Floreat.

	Mean temperature (°C)			
Dry-down group/trial year	2016	2017	2018	2019
1	16.4	17.4	17.7	17.8
2	16.8	18.4	18.0	
3	17.4	20.6	19.2	
4	18.2	22.4		
5	19.3			
6	19.4			
7	19.0			
8	20.7			
9	21.8			
10	21.3			
DD period temp change (°C/day)	0.09	0.18	0.13	−0.02
Vegetative phase rainfall (mm)	463.2	540.4	444.6	461.8
Veg phase mean temp (°C)	13.3	15.0	14.9	15.2

Vegetative phase rainfall and temperature calculated up to the start of the dry-down phase.

**Figure 2:**
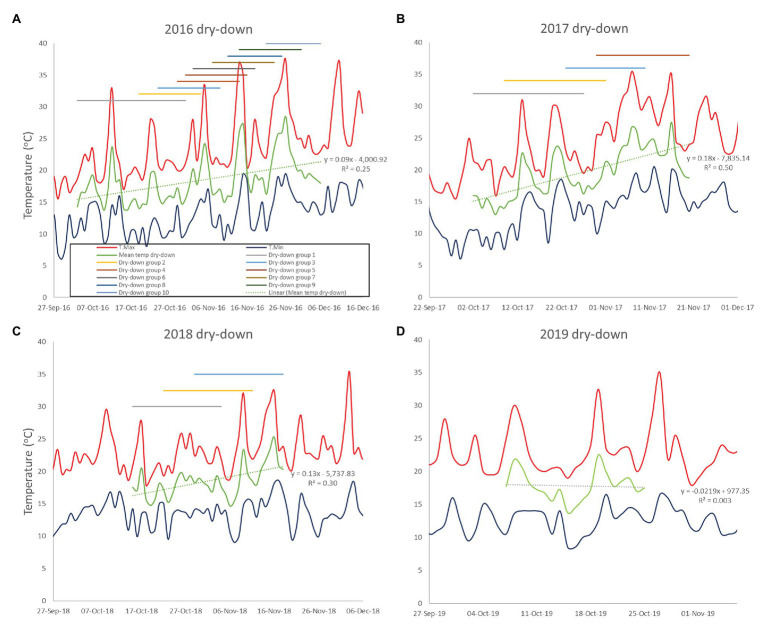
Minimum and maximum temperatures during the dry-down phases of the 2016–2019 CSIRO rainout shelter trials **(A-D)**. The daily mean temperature of the combined dry-down period is shown as the durations of the separate dry-down phases.

In the 2016, trial all plots were planted on 9th June. Large phenological differences among accessions meant that 10 separate dry-down groups were required to initiate the water deficit treatment from the onset of pod filling (Figure 2A; Table 2). The combined dry-down period in the 2016 trial was characterized by gradually rising temperatures punctuated by temperature spikes at approximately 10-day intervals such that later groups experienced more terminal drought stress than earlier groups (Figure 2A; Table 2).

To ensure consistent terminal drought stress later trials used a combination of vernalization (4 weeks at 4°C) and staggered sowing dates to make the onset date of water deficit as uniform as possible. To this end, the 2017 and 2018 trials were sown over six staggered occasions according to phenology from 7th June to 12th July and 18th June to 9th July, respectively. Despite our attempts to synchronize reproductive phases, in the 2017 trial, the onset of water deficit was still staggered across four groups starting from 2nd to 30th October. Over this period, there was a linear temperature increase punctuated by high temperature spikes driving sequentially increasing terminal drought stress in later dry-down groups (Figure 2B; Table 2). The 2018 staggered sowing was more effective with the onset of water deficit staggered across three groups at weekly intervals from 15th to 29th October, with smaller temperature differences driven by a weaker linear temperature rise (Figure 2C; Table 2). Nevertheless, isolated hot days (maxima > 30.0°C) in early-mid November 2018 added stress to the end of the dry-down period in groups 2 and particularly 3 (Figure 2C).

In the 2019, trial sowing dates were widened even more to ensure reproductive synchrony. Fourteen staggered sowing dates over 37 days were used in each of three staggered starting dates (3rd June, 10th June, and 17th June) to grow three times as many pots as required for the experiment. In early October, plants with similar pod development were selected for all treatments and moved into the final split plot configuration such that the 2019 trial contained only a single, synchronous dry-down group exposed to a similar temperature range as previous early groups (Figure 2D; Table 2). Unlike previous water deficit treatments, the 2019 TD treatment was irrigated on day 2 (receiving 50% of the water used calculated individually for each pot) to extend the dry down period because of a temperature spike (Figure 2D). Thereafter, no water was added to the TD treatment, as in the 2017–2018 experiments.

### Observations

Phenology was measured as described previously. Plant maturity (defined as 95% of pods ripe) was recorded in 2016 and 2018.

Plants were bagged prior to pod maturity to prevent seed loss during shattering. Subsequently, plants were counted and harvested, total biomass, seed weight, and number recorded. Seed size and harvest index were calculated from this data. A TD stress index was calculated based on the percentage of the well-watered value (Bouslama and Schapaugh Jr., 1984).

Reproductive water-use was measured by weighing pots at 2-day intervals after the onset of the terminal drought. Final pot weights were used to calculated plant available water (PAW) at each weighing. This data were used to calculate reproductive WUE, PAW% and transpiration per unit time, expressed in days, thermal time or cumulative evapotranspiration. In 2018 and 2019, water-use was measured in both water regimes.

### Wild and Domestic Developmental Differences Over the Lifecycle (Exp 5)

To better understand behavior of wild and domestic *Cicer* under contrasting water supply, it was necessary to develop an understanding of developmental differences between the groups. To this end, we designed a glasshouse pot trial measuring above and below-ground dry matter partitioning and their effects on water extraction and WUE. To describe the effects of domestication independently of phenology, we studied plant development throughout the lifecycle in balanced early and late phenology subsets of wild and domesticated *Cicer* (Table 3). These were grown as single plants in 120 × 16 cm split pots filled to 100 cm with Gingin loam using an RCBD (*n* = 3), with extra replication (*n* = 3 per harvest) allowing for destructive harvests at 35, 70, and 105 days and a final harvest at physiological maturity (155–170 days). The experiment was sown on June 20 2017 to coincide with the normal Mediterranean winter growing season after 4 weeks of vernalization at 4°C.

**Table 3:** Early and late phenology subsets of domestic (*C. arietinum*) and wild *Cicer* (*C. echinospermum* and *C. reticulatum*) selected for studying developmental differences across the lifecycle.

Species	Early	Late
*Cicer arietinum*	ICCV 93929 (Indian desi cv.)	Almaz (Australian kabuli cv.)
*Cicer echinospermum*	S2Drd_061	Ortan_066, Cermi_063
*Cicer reticulatum*	Besev_066	Sirna_060, Sirna_063

At sowing, the soil was filled to field capacity and water-use monitored at approximately 2-day intervals throughout the growing season. This was done by applying a known amount of water and calculating daily water-use by subtracting the overflow emerging from a tube at the base of each pot. To minimize evaporation, the soil surface layer was covered by plastic beads to a depth of 5 cm. Cumulative water-use curves were generated by fitting logistic functions to the daily summed water-use (see Statistical Analysis). Water inputs were matched to water use to avoid over-filling. Water-use data were used to calculate WUE of above ground biomass at each destructive harvest and reproductive WUE using pod weights at final harvest. Reproductive WUE was calculated on cumulative water-use between the start of podding and final harvest (as in the rainout shelter experiments), biomass WUE was based on water-use since the start of the experiment and each destructive harvest. Phenology observations were made on all pots throughout the growing season as described in the rainout shelter experiments.

Water inputs were stopped for those pots slated for destructive harvest 1 week before each harvest date to reveal water extraction profiles along the soil column. The above ground biomass was removed, processed into vegetative and reproductive tissue, and leaf area measured (only until day 105 because leaves were shed by physiological maturity). The split pots were carefully divided in two without disturbing the soil column, which was then separated into 20 cm segments (0–20, 20–40, 60–80, and 80–100 cm). Soil in each segment was sub-sampled, fresh and dry weights recorded (after 48 h oven drying at 60°C) to calculate the relative water content [(fresh-dry weight)/dry weight]. Roots were carefully washed out of each soil segment, sieved, and dried. The data from each segment were used to explore relationships with depth (see Statistical Analysis). Root weights from each segment were also summed to provide a total root biomass, which was used to calculate root index (percent of total biomass attributable to roots), shoot to root ratio, vegetative, and total biomass (sum of above and below ground biomass). Below ground data were only available for the first three harvest dates because by physiological maturity the roots had started to decay.

### Statistical Analysis

Genstat (V20) was used for all statistical analyses. Nested ANOVA and regression models were used to partition variance between species, between collection sites within species and finally between accessions within collection sites within species. Wild and domestic differences were analyzed using orthogonal contrasts. The same nested approach was used in the lifecycle development study, except that accessions were nested within phenology categories within species, rather than collection sites within species.

Replications were fitted within water regime in the RCBDs used in 2016–2017, where the water regime treatments were established on separate contiguous areas of the rainout shelter. In the 2018–2019, split plot designs water regime was treated as the main plot, accessions as the subplot. The lifecycle development study was analyzed as a standard RCBD using reps as blocks.

Linear and non-linear {exponential, *Y* = *a* + *b*r^X^; logistic, *Y* = *a* + *c*/[1 + e^−b(X − m)^]} regression was used to model changes in PAWC and biomass over time, and root growth and water extraction over depth in the development study. These regression models were chosen to best fit the trend in the data, producing residual plots with normally, and independently distributed errors. Changes in PAWC and leaf area over time, and root growth over time and depth were exponential. Water-use, vegetative matter, and total biomass followed logistic patterns over time, while WUE over the growing season was well-modeled by quadratic regression.

Residual plots were used throughout to identify outliers and confirm that errors were normally and independently distributed.

Correlation-based principal components analysis (PCA) was used to integrate the results using two-way accession by water regime means and curve parameters generated by the analyses described above.

## Results

### *Cicer* Species Responses to Terminal Water Deficit: Productivity, Phenology, and Water-Use

Nested ANOVA demonstrated consistent patterns among traits across years. In those phenological observations taken prior to the onset of water deficit treatments, the largest differences occurred between species, followed by collection sites within species, and finally accessions within collection sites (*p* < 0.001 for all). Observations made after the imposition of water deficit tended to follow a similar pattern, albeit with significant interactions with water regime.

Domestic chickpea was characterized by a consistently earlier phenology than wild *C. reticulatum* and *C. echinospermum* (Table 4). Moving from a common (2016) to a staggered sowing date (2017 onward) to try to synchronize the reproductive phase shortened the vegetative phase, particularly in the 2019 trial, based on the widest combination of sowing dates (Table 4).

**Table 4:** Domestic (*C. arietinum*) vs. wild (*C. echinospermum* and *C. reticulatum*) phenology (flowering and podding means) in the 2016–2019 dry-down experiments.

Exp yr	*Cicer arietinum*	*Cicer echinospermum*	*Cicer reticulatum*	LSD^*^	Pval: wild vs. dom
**Flowering**					
2016	90	117	121	1	<0.001
2017	67	93	92	4	<0.001
2018	78	97	92	2	<0.001
2019	68	77	78	2	<0.001
**Podding**					
2016	109	127	130	1	<0.001
2017	82	103	102	3	<0.001
2018	88	105	101	2	<0.001
2019	79	86	88	5	<0.001

* Least significant difference (LSD).

**Figure 3:**
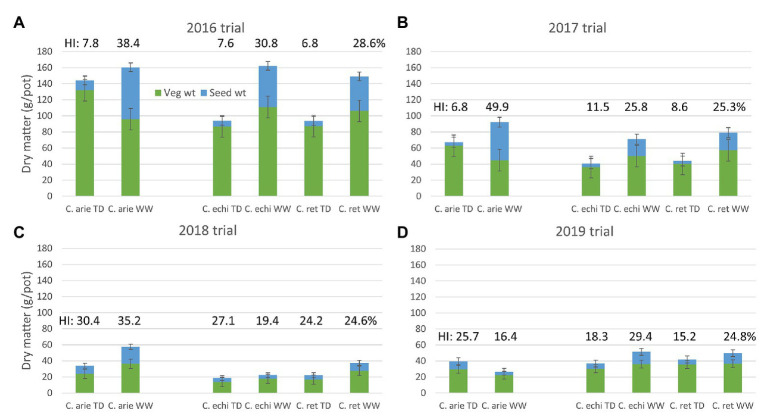
Domestic (C. arie, *C. arietinum*) vs. wild *Cicer* (C. echi, *C. echinospermum*; C. ret, *C. reticulatum*) productivity (total biomass, vegetative, and seed weight) under well-watered (WW) and terminal drought (TD) treatments in the 2016–2019 rainout shelter trials **(A-D)**. Error bars represent 1 LSD, the proportion of vegetative to seed weight represents harvest index (HI), values included above columns.

The reduction of the vegetative phase had ramifications on biomass production. Total biomass decreased as the vegetative phase was shrunk from 2016 to 2019 (Table 4; Figure 3). Despite these scale changes, water deficit consistently reduced above ground biomass production in all trials, albeit with some differences between species. In 2016 and 2019, wild *Cicer* was more responsive to the WW treatment than domestic chickpea (*p* < 0.001), in 2017, there was no difference (*p*_diff_ = 0.390), while in 2018, *C. echinospermum* was uncharacteristically unresponsive (Figure 3). These interactions notwithstanding, domestic chickpea tended to accumulate more above ground biomass than wild *Cicer* on most occasions (Figure 3, TD: 2016–2018; WW: 2017–2018).

Under terminal drought, wild and domestic *Cicer* had consistently similar reproductive investment (Figure 3: harvest index and seed weight). Indeed, only the 2019, TD treatment plants exhibited a significant harvest index difference in favor of domestic chickpea (*p* < 0.05), while seed weights were similar. However, there were clear domestic/wild differences in the reproductive response to the additional irrigation provided by the WW treatment. In 3 of the 4 trial years (2016–2018) harvest index and seed weight increased more in domesticated than in wild *Cicer* (Figure 3; *p*_diff_ = 0.013–<0.001) in response to WW treatment irrigation.

**Figure 4:**
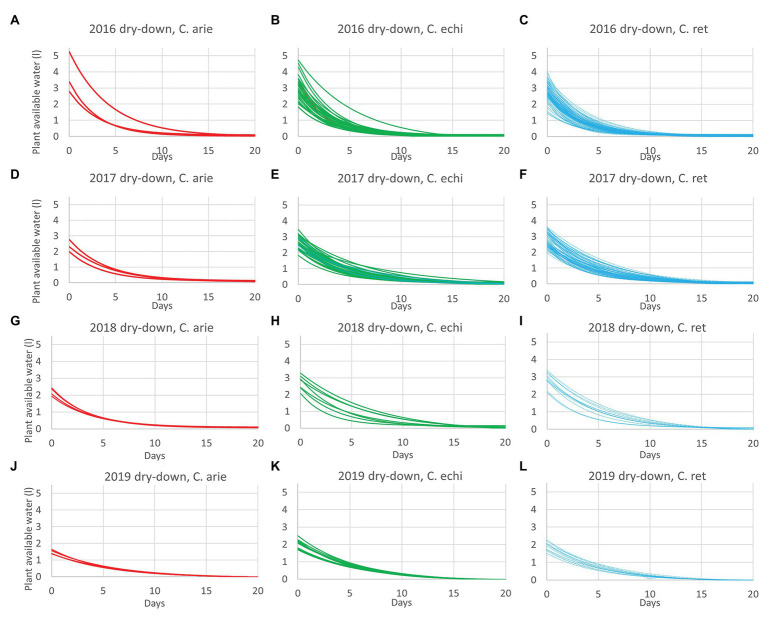
Domestic (C. arie, *C. arietinum*, 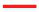) vs. wild *Cicer* (C. echi, *C. echinospermum*, 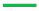; C. ret., *C. reticulatum*, 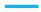) water-use after the onset of terminal drought in dry-down experiments from 2016 to 2019 **(A-L)**. Exponential curves fitted for accessions within species captured 89.6–96.9% of variance.

The reduction of vegetative phase length and total biomass production over years had implications on plant reproductive water-use, measured gravimetrically during the dry-down cycle. This is clearly indicated by reduced water extraction from 2016 to 2019 in all species (Figure 4; Table 5). Nevertheless, there were remarkably consistent, contrasting water-use patterns between domestic and wild *Cicer* across all years (Figure 4). Thus, domestic chickpea tended to extract less water in the dry down cycle than either wild species, as indicated by significant *y* intercept differences (Figure 4; *p* < 0.001) in all years, captured by the exponential curve parameter B (see Table 5 for raw water uptake values). Surprisingly, the exponential rate of water-use (parameter *R*) was higher in chickpea than in wild *Cicer* from 2017 to 2018 (*p* < 0.05). These curve parameter differences are clearly evident across years in Figure 3, with chickpea varieties forming a tight cluster at the lower end of the water extraction range (except for ICCV 93929 as a high extracting outlier in 2016 only). Conversely, the two wild species were characterized by a wider range of water-use curves among accessions, corresponding to higher mean values than in domestic chickpea (Figure 4).

**Table 5:** Domestic (*C. arietinum*) vs. wild (*C. echinospermum* and *C. reticulatum*) plant water uptake and reproductive water use efficiency (WUE) in the 2016–2019 dry-down experiments.

Treat/yr	*Cicer arietinum*	*Cicer echinospermum*	*Cicer reticulatum*	LSD	Pval: wild vs. dom
**Plant water uptake (L)**					
2016 TD	3.76	3.02	2.78	0.33	<0.001
2017 TD	2.45	2.66	2.79	0.30	0.065
2018 TD	2.24	2.79	2.77	0.29	<0.001
2018 WW	7.20	7.91	9.23	1.35	0.033
2019 TD	2.10	2.70	2.40	0.22	<0.001
2019 WW	6.80	7.70	7.50	0.62	0.006
**Reproductive water use efficiency (seed weight g/L)**					
2016 TD	4.0	2.8	2.6	0.8	0.002
2017 TD	1.9	2.2	1.5	0.6	0.947
2018 TD	4.9	2.2	2.2	1.1	<0.001
2018 WW	3.0	0.9	1.1	0.5	<0.001
2019 TD	4.9	2.5	2.7	0.7	<0.001
2019 WW	0.6	2.0	1.9	0.4	<0.001

TD, terminal drought; WW, well-watered reproductive phase; LSD, least significant difference.

In 2018 and 2019, water-use was also measured in the WW treatment. ANOVA was dominated by very large wild and domestic differences (*p*_diff_ = 0.005–<0.001), without water regime interaction (*p*_diff_ = 0.2110.298). This is important because it indicates that while the wild *Cicer* species extracted more water under terminal drought, they also consistently used more water under the WW treatment (Table 5).

### Integrating the Results With Multivariate Analysis

**Figure 5:**
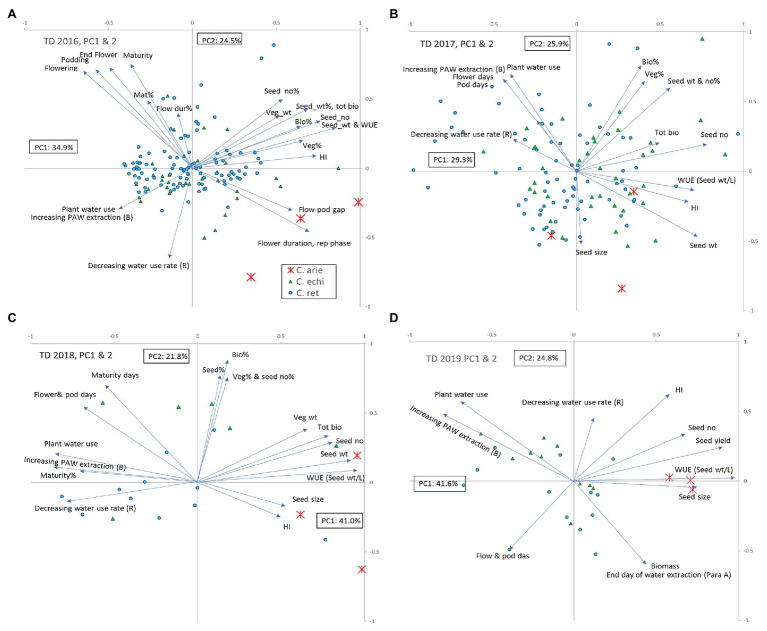
Principal components analysis of observations recorded in the terminal drought treatments in the 2016–2019 water deficit trials **(A-D)**. Vectors represent factor loadings in PC1 and PC2, markers represent accession scores classified by species: ✱, C. arie, *C. arietinum*; ▲, C. echi, *C. echinospermum*; ●, C. ret, *C. reticulatum*. Bio, biomass; HI, harvest index; rep, reproductive; veg, vegetative; WUE, water use efficiency; %, TD stress index: percentage of the well-watered value recorded in the terminal drought treatment.

Principal components analysis integrated the observations made in the TD treatment, capturing 55–66% of variance in two components in the ordinations performed for the 2016–2019 trials (Figure 5). Wild and domestic *Cicer* were consistently separated by phenology, water extraction, and WUE (Figure 5; Table 5; all years), and to a lesser extent rate of use (2017–2019). Productivity traits (seed weight, biomass, etc.,) were consistently closely associated with harvest index and negatively correlated with water extraction and WUE. Thus, high yielding plants had high harvest index and WUE but extracted less water during the reproductive phase dry-down than low yielding plants. In 2 of the 3 years where TD stress indices were calculated (2016–2017) plants with high TD productivity achieved a high proportion of their WW productivity, that is to say they were less responsive to the more benign WW treatment than poorly yielding plants [TD stress indices were not calculated in 2019 because domestic chickpea performed so poorly in the WW treatment (Figure 3D) rendering the wild comparison meaningless].

Principal components analysis confirmed the specific distinctions described earlier that wild *Cicer* tends to have later phenology, lower productivity, and greater water extraction coupled with lower maximum use rates than domestic chickpea. However, PCA also demonstrated considerable within species variation in these traits, particularly in 2016–2017, when a much larger wild cohort was investigated. In both these years, some *C. reticulatum* and *C. echinospermum* accessions had similar, or greater productivity, WUE and harvest index than the most productive domestic chickpea (Figures 5A,B). The ordinations for the WW treatment were remarkably similar (data not presented). Wild and domestic *Cicer* were separated by phenology, productivity, harvest index, and WUE (measured in 2018 only). As in the TD treatment, seed yield was positively correlated with harvest index and WUE, and negatively correlated with phenology. With very few exceptions, domestic chickpea was more productive than wild *Cicer* in the WW treatment, associated with greater reproductive investment and WUE.

### Wild and Domestic Developmental Differences

To independently test the effects of phenology and “wildness” on above and below-ground dry matter vegetative and reproductive partitioning, and their effects on water extraction and WUE, we studied plant development throughout the lifecycle in balanced early and late phenology subsets of wild and domestic *Cicer* grown in the glasshouse.

**Figure 6:**
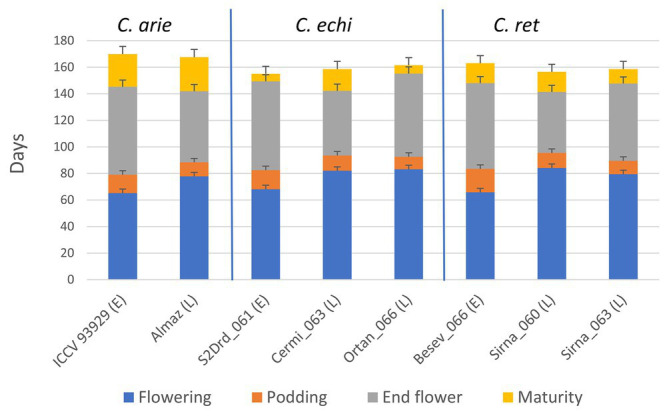
Life cycle phenology in early (E) and late (L) flowering domestic (C. arie, *C. arietinum*) and wild *Cicer* (C. echi, *C. echinospermum*; C. ret, *C. reticulatum*) selected to study the role of “wildness” and phenology on plant development and water use under *ad libitum* water over the growing season. LSD bars (least significant difference) are presented for each trait individually.

This experimental approach set vegetative phase limits within and between species. Figure 6 shows consistent phenology category differences between species for flowering and podding, not for end of flowering and particularly maturity. Thus, our phenology categories reliably determined the length of the vegetative phase and the onset of reproduction (e.g., short and early vs. longer and later) with no, or very minor species differences (Figure 6). However, domestic chickpea matured approximately 10 days later than wild *Cicer* (Figure 6; *p* < 0.001), with important flow-on effects on the length of the reproductive phase. While the reproductive phase was consistently 9–22 days longer in the early compared to late phenology groups, the domestic reproductive phase was 9–20 and 12–15 days longer than wild in the early and late categories, respectively (Figure 6).

**Figure 7:**
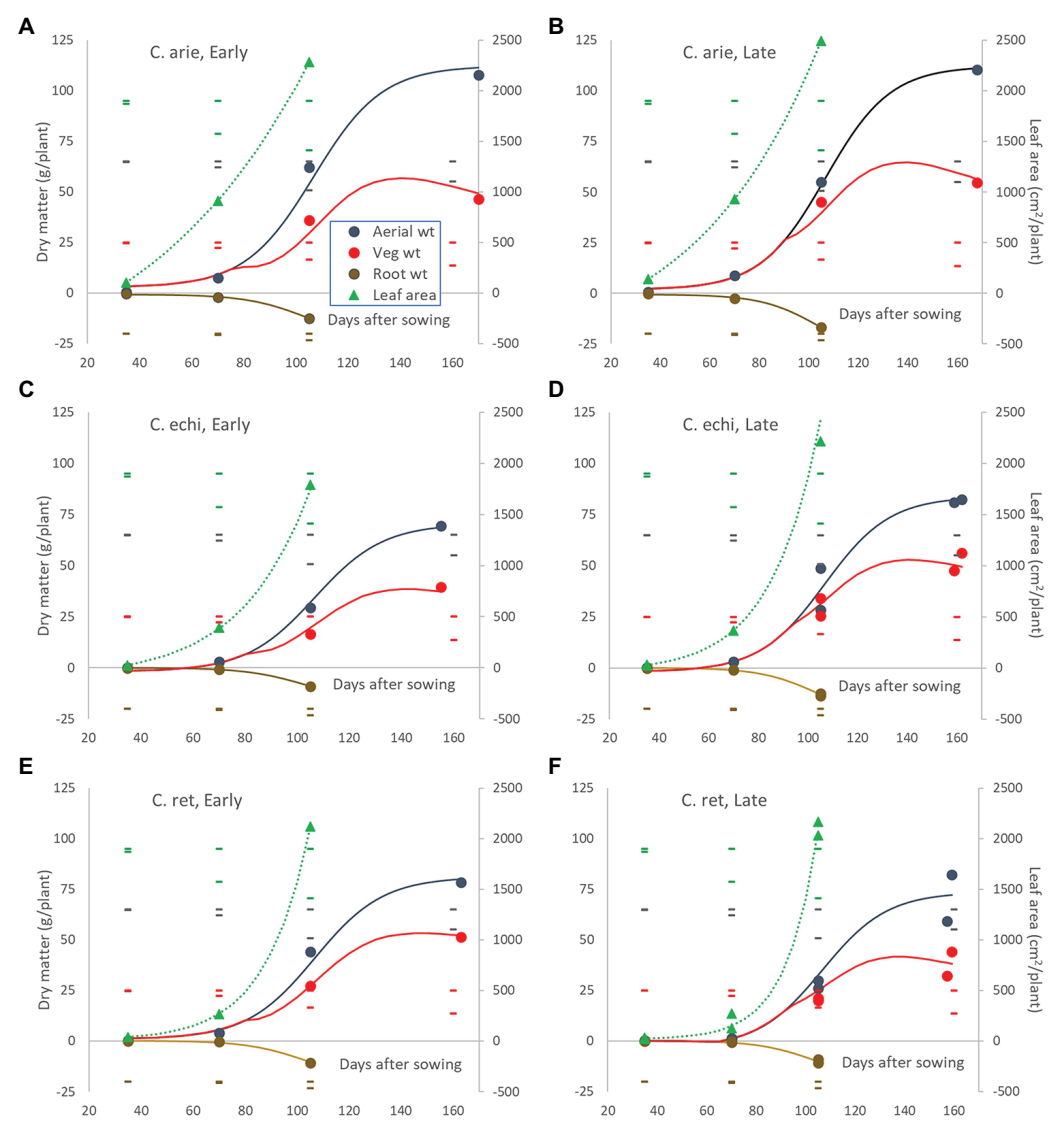
Leaf area and above‐ and below-ground dry matter partitioning over the growing season in early and late flowering domestic **(A,B)** and wild *Cicer* **(C–F)**. Color coded markers, fitted curves and daily LSD bars (least significant difference) are presented for leaf area (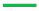), root weight (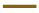), vegetative matter (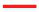), and total aerial biomass (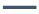). Point values represent accession means from destructive harvests at 35, 70, 105, and 155–170 days after sowing. Logistic and exponential curves fitted for species/phenology categories captured 92.6–96.3% of variance, accounting for all differences between accessions. The area between the vegetative matter and aerial biomass curves represents pod weight. Root weights are presented as negative values to facilitate visual root-shoot comparisons over time.

These species and phenology category differences played out in plant development. Polynomial contrasts in nested ANOVA showed strong linear and quadratic interactions between species and phenology categories within species for most measured traits (*p* < 0.001). Because of developmental lag phases and ceiling values in most of these plant structures, non-linear logistic and occasionally exponential regression was generally more appropriate (Figure 7), confirming the significant interactions indicate by ANOVA.

The early harvests (35 and 70 days) were dominated by wild vs. domestic *Cicer* differences. Domestic chickpea had significantly greater early vigor than wild *Cicer* (*p* < 0.001), producing far greater leaf area, root, and shoot mass at 35 and 70 days, accounting for almost all significant differences (Figure 7). The only exception was at 35 days where the late kabuli variety Almaz had much greater biomass than the early desi ICCV 93929, a difference that disappeared by day 70. Thus, the early-mid vegetative phase growth rates were far higher in domestic chickpea than wild *Cicer*, particularly for leaf area (Figure 7).

**Figure 8:**
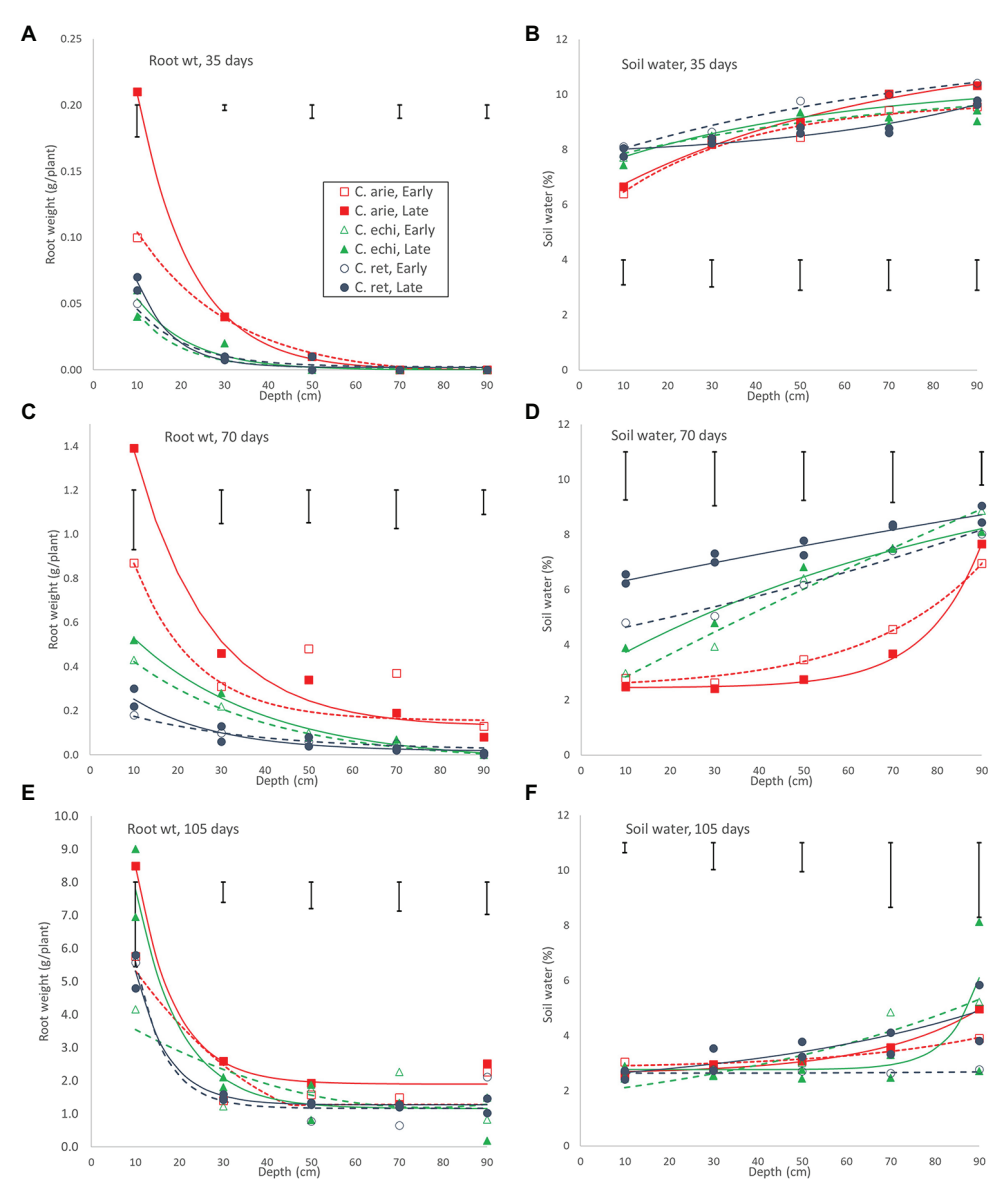
Root development and water extraction over depth over the growing season in early and late flowering domestic (C. arie, *C. arietinum*) and wild *Cicer* (C. echi, *C. echinospermum*; C. ret, *C. reticulatum*). Point values represent accession means from destructive harvests at 35 **(A,B)**, 70 **(C,D)**, and 105 days after sowing **(E,F)**. Error bars represent LSD values for individual harvests. Exponential curves fitted for species/phenology categories captured 86.8–97.4% and 37.6–83.1% of variance for root weight and water extraction, respectively.

These wild-domestic early vigor differences were reflected in root development and water extraction down the soil profile. Root mass and water extraction decreased curvi-linearly with depth in all species (Figure 8). At 35 days, domestic chickpea (particularly the kabuli cultivar, Almaz) had far greater rates of root weight decline and water extraction over depth than wild *Cicer* (*p* < 0.001), driven by massive differences in the 0–20 cm soil layer, disappearing by 40–60 cm (*p*_diff_ = 0.334), the limit of root exploration (Figure 8A). Interestingly, water extraction of the two chickpea cultivars was similar, despite their differences in surface root production. Domestic water extraction was greater than wild in the upper soil layer (*p* < 0.001) but not at greater depths (Figure 8B; *p*_diff_ = 0.150). At 70 days, wild and domestic differences still dominated, albeit these were becoming smaller in terms of root distribution (Figure 8C). Root decline rates over depth were similar across all accessions, regardless of species or phenology category, except for Almaz (*p*_diff_ < 0.001) with its high surface layer root mass. Nevertheless, there were significant intercept differences between the desi cultivar ICCV 93929 and most of the wild *Cicer* (*p*_diff_ = 0.127–0.001), reflecting root mass differences at most depths (Figure 8C). These wild-domestic differences had a large impact on water extraction down the soil profile (Figure 8D). Domestic chickpea depleted water from the top three soil layers, leaving progressively more water in the remaining two soil layers, captured by a similar upward trending quadratic curve in both the early desi and late kabuli varieties. Conversely, the wild *Cicer* water extraction curve was much more linear over depth, leaving significantly more residual water in all layers (*p* < 0.001).

Day 70 marked the end of the lag phase when all species showed rapid growth rates for above and below ground biomass, and new species differences and phenology category by species interactions were emerged. Thus, the early specific differences in root growth rates disappeared during the rapid growth phase, while phenology category by species interactions emerged, indicated by higher growth rates in the late compared to the early *Cicer arietinum* (*p*_diff_ = 0.015) and *C. echinospermum* (*p*_diff_ = 0.027), but not *C. reticulatum* (Figure 7, *p*_diff_ = 0.733). This pattern was clearly evident in the root distribution over depth, with steeper declines in late compared to early *C. arietinum* (*p*_diff_ = 0.065) and *C. echinospermum* (*p*_diff_ = 0.002), but not *C. reticulatum* (Figure 8E, *p*_diff_ = 0.259). Accordingly, there were no specific differences in water extraction by day 70, with a common, relatively flat curvi-linear response (Figure 8F, *p*_diff_ = 0.467–0.708), leaving only minor variety within phenology category differences. Above-ground vegetative biomass growth curves were clearly logistic across the range of harvest dates (Figure 7). As with root growth, there were no consistent specific differences.

In terms of leaf area growth species differences trumped phenology interactions, but now the wild *Cicer* had a higher rate of leaf area production than domestic chickpea (*P*_diff_ = 0.048), such that by 105 days there were no specific differences in leaf area (Figure 7, *P*_diff_ = 0.192).

**Figure 9:**
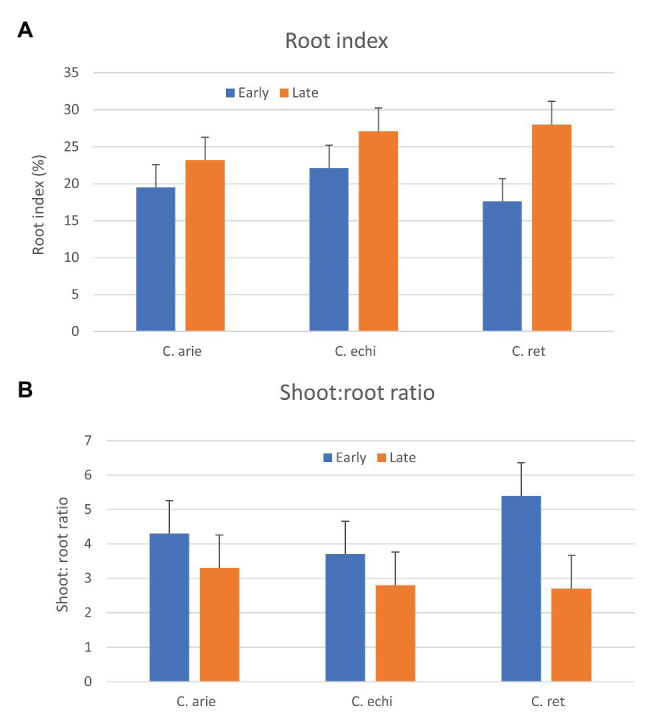
Relative above and below-ground investment (**A**, Root index; **B**, shoot to root ratio) in early and late flowering domestic (C. arie, *C. arietinum*) and wild *Cicer* (C. echi, *C. echinospermum*; C. ret, *C. reticulatum*). Error bars represent LSD values.

Phenology and “wildness” were both important in the relative above‐ and below-ground dry matter partitioning, indicated by strong species by phenology category interaction (*p* < 0.001). While later types of all species invested more heavily in roots, as indicated by higher root indices and lower shoot:root ratios (Figure 9), the contrast was much stronger in wild than domesticated *Cicer*, particularly *C. reticulatum*. Thus, late wild *Cicer* have a much greater root index than late domestic chickpea, while there were no specific differences among the early group. Interestingly, this pattern was evident already at 35 days after sowing, well before the start of flowering, suggesting that these differences are not explained by differences in the vegetative phase length.

Pod growth rates also showed strong species by phenology category interaction (*p* < 0.001). Chickpea rates were considerably higher than wild, while late types of all species tended to fill pods at higher rates than early types, reaching similar final pod weights at maturity despite a later podding onset (Figure 7). Pod growth rate differences between early and late types were larger in *C. reticulatum* than in *C. arietinum* and *C. echinospermum*, accounting for the significant interaction. Ultimately at maturity, reproductive investment (harvest index) was far greater in domestic than wild *Cicer* (*p* < 0.001), and greater in early compared to late *C. arietinum* and *C. echinospermum*, but not *C. reticulatum*. The combination of rapid pod growth rates and high reproductive investment was responsible for higher rates of aerial biomass production in domestic vs. wild *Cicer* over the growing season (*p* < 0.001), with no differences between phenology categories within species (Figure 7). The final areal biomass values at maturity largely reflected these rate differences: domestic larger than wild (*p* < 0.001), late *C. echinospermum* larger than early, whereas the opposite was the case for *C. reticulatum* (*P*_diff_ = 0.05).

**Figure 10:**
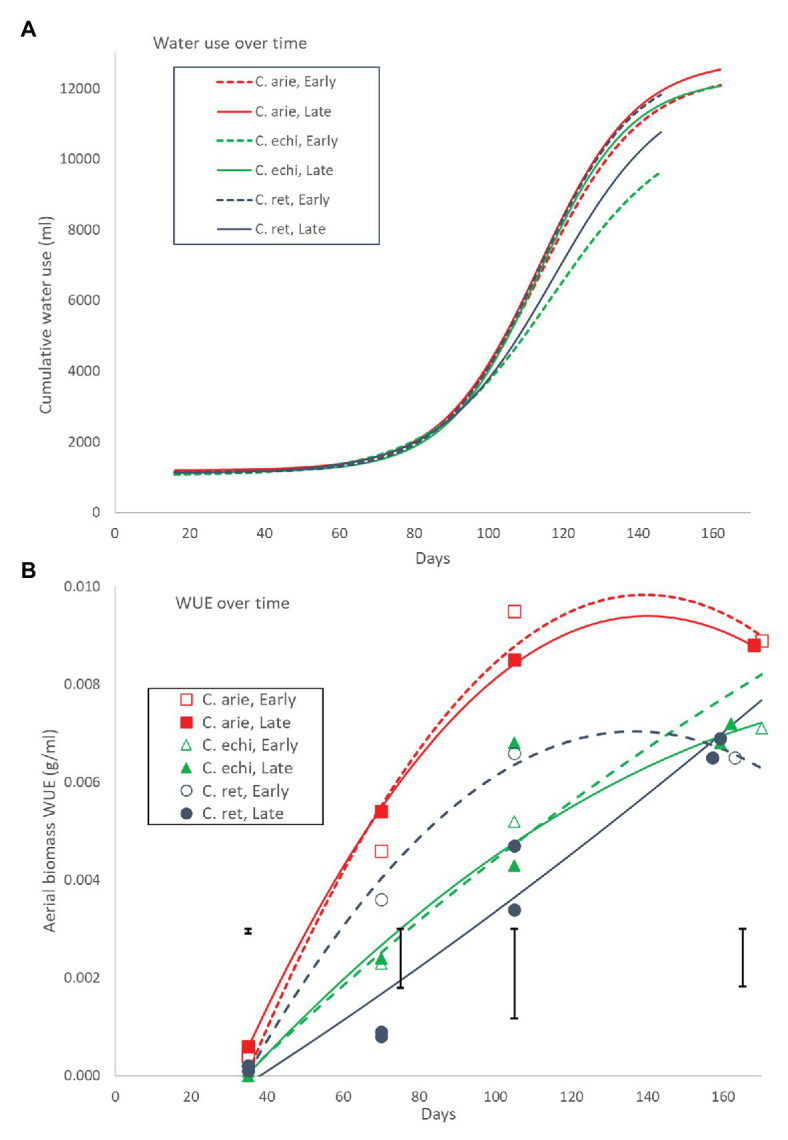
Water-use **(A)** and WUE **(B)** over the growing season in early and late flowering domestic (C. arie, *C. arietinum*) and wild *Cicer* (C. echi, *C. echinospermum*; C. ret, *C. reticulatum*). The non-linear logistic and linear quadratic curves in **(A)** and **(B)** account for 98.0 and 92.1% of variance, with significant species/phenology category differences for all parameters. Point values in **(B)** represent accession means from destructive harvests at 35, 70, 105, and 155–170 days after sowing. Error bars represent LSD values for individual harvests.

Plant water-use throughout the growing season followed a logistic curve largely, but not totally mirroring biomass production (Figure 10A). Daily water-use rates remained flat for the first 70 days with minor wild vs. domestic differences (12 vs. 13 ml/day, *P*_diff_ = 0.014), turning sharply at 90 days, and peaking at 111 days. Moreover, the species/phenology characteristics described earlier also played out in the water-use curves: later *C. arietinum* and *C. echinospermum* used more water [parameter C (curve maximum value), *p* < 0.001] at higher rates (logistic growth rate *k*, *p* < 0.001) than early types, while the opposite was the case for *C. reticulatum* (Figure 10A; *p* < 0.001). Nevertheless, the wild and domestic curves were remarkably similar, given differences in their aerial biomass. This is reflected in large wild vs. domestic differences in WUE across the life cycle (Figure 10B). While all species became more efficient in their water-use for aerial biomass production over time, the rate of increase was much larger in domestic vs. wild *Cicer* (Figure 10B; *p* < 0.001). Domestic WUE was larger than wild (*p* < 0.001) at every point sampled throughout the lifecycle. Moreover, there were important differences in the shape of the response. In domestic chickpea (and early *C. reticulatum*), the rise in WUE over time was curvi-linear, peaking approximately 2/3 of the way through the life-cycle, whereas the response in the remaining wild *Cicer* groups was much linear, peaking at maturity (Figure 10B). Nevertheless, at maturity the domestic WUE remained higher than wild (Figure 9B; *p* < 0.001), accounting for all phenology/species category or variety differences (*p*_diff_ = 0.901 and 0.561).

## Discussion

Our work shows that domestication has ramifications throughout the entire lifecycle in this *Cicer* example, but that domestication as a promotor of acquisitiveness is not particularly helpful to distinguish wild and domesticate. While domestic chickpea was indeed much more responsive to resource-rich conditions, and wild and domestic differences minimized under terminal drought (similar to Matesanz and Milla, 2018), these differences were not driven by greater resource acquisition in domestic chickpea. On the contrary, wild *Cicer* was able to extract more water under water deficit, but also used more water when it was freely available. Nor were there consistent wild-domestic growth rate differences across all plant organs over time, as would be predicted if wild and domestic occupied different ends of the stress tolerator-competitor continuum (Grime, 2006). While early vegetative growth and water extraction was much more rapid in domestic compared to wild *Cicer*, this is likely to be a function of greater early vigor, presumably a by-product of selecting large seed sizes. This is a common phenomenon in both domesticated cereals (Evans and Dunstone, 1970) and grain legumes (Berger et al., 2017), and underlined in this example by the early wild and domestic growth differences, and between desi and the much larger seeded kabuli type within domesticated chickpea. The fact that these growth rate differences disappeared over the growing season highlights the need to study development over the entire lifecycle. Indeed, in the late vegetative phase, as growth rates became exponential, rates of leaf area expansion were considerably greater in wild compared to domestic *Cicer*.

Clearly, the slow-wild/fast-domestic dichotomy is not supported in this *Cicer* example. Unpacking the stress tolerator-competitor continuum (Grime, 2006) in an agricultural context indicates why this may be. The triangle of Grime (2006) suggests that acquisitive traits such as rapid growth rates above and below ground are selected for in resource-rich environments, where there is strong competition for these resources (the “use it or lose it” scenario, see examples in Grime, 1988, 2006). Conversely, this strategy is risky and maladaptive in resource-poor environments, where periods of stress have to be tolerated. These environments select for slow, resource efficient growth, and stress tolerating physiology (Grime, 2006) that is strongly expressed in extremophiles such as desert cacti and succulents (Ehleringer and Mooney, 1983), and is less relevant to annual plants and agriculture (Berger et al., 2016). The lifecycles of annual plants and most agricultural crops balance stress escape (the ruderal strategy, third apex in the triangle of Grime, 2006) against acquisitiveness. Although well-managed fertile fields are likely to represent a more resource-rich environment for crops than the natural systems in which their wild progenitors evolved, this is unlikely to have selected for greater acquisitiveness (aka competitive capacity) because of the disparate selection pressures imposed by the two systems. Whereas wild plants are selected as individuals, crops are grown and selected as populations rather than as single plants. This has selected for crops that are poor competitors as individual plants, lifting the productivity of the community as a whole, rather than maximizing the fitness of the individual (Donald, 1963, 1981; Reynolds et al., 1994). This is exactly what we see in our results, where the wild *Cicer* consistently extract and use more water (which would otherwise be lost to their competitors) than their domesticated counterparts. In fact, far from being parsimonious and efficient stress tolerators, the wild *Cicer* seems to be profligate competitors compared to domestic chickpea. Indeed, the poor competitor aspect of chickpea water-use is being exploited in the development of cultivars for short season, stored soil moisture systems in the semi-arid tropics. ICRISAT is promoting the use of cultivars that use less water in the vegetative phase, leaving more residual soil water for seed filling (Zaman-Allah et al., 2011), a scenario that is difficult to envisage occurring in the natural world of inter-plant competition.

Instead of greater acquisitiveness *Cicer* domestication appears to have selected for greater reproductive efficiency, whether measured in terms of dry matter allocation or WUE. This is particularly apparent under high input conditions, where domestic chickpea is far more responsive than wild in terms of pod growth rates and harvest index. Conversely, these studies provide evidence of greater vegetative investment by the wild *Cicer* in terms of later phenology, higher rates of leaf area production, and greater relative investment in roots, particularly in late types. This combination facilitates greater water extraction under terminal drought, but also allows greater water-use when it is freely available. Here, there are interesting parallels with the Old World lupins, where there are similar wild-domestic differences in reproductive and vegetative investment, and flow-on effects on water use (Berger and Ludwig, 2014; Berger et al., 2020). The cereals may be more conservative in terms of relative dry matter partitioning (Wacker et al., 2002; see Wang et al., 2017 for counter-indication), but do show a similar trend in tillering along the domestication series. Thus, post anthesis tillering decreases in domestic tetra and hexaploid wheat compared to the diploid wild progenitors (Evans and Dunstone, 1970). Interestingly, similar phenology-reproductive investment trade-offs were seen in wild emmer × durum RIL populations, highlighted by the remarkable similarity in the ordinations presented by Peleg et al. (2009) and those in this manuscript. These patterns align well with competitor-ruderal continuum of Grime (2006). By selecting for earlier phenology to fit crops into a time delimited production system, domesticated crops took on ruderal attributes such as increased harvest index, and seedling establishment was improved by greater early vigor, associated with large grain size (Berger et al., 2017). This is a productive and efficient, but risky reproductive strategy that works in agriculture where the crop is protected from grazing, disease, and competition, circumstances that do not pertain in the wild. Life is less certain for wild *Cicer*, and flexibility appears to be more important than reproductive efficiency over the long term. Our field observations support this idea: we have seen wild accessions re-growing from the base after Ascochyta has killed the aerial plant parts, or after grazing and also after very late rains when the plant had seemingly matured, leaving only shattered pods, and dry straw.

Apart from differences in vegetative and reproductive dry matter allocation, domestic water-use was also more efficient than wild. In cereals, this has been attributed to greater harvest index (Wang et al., 2017). While our later season results confirm this idea, the fact that this trend was already apparent in the vegetative phase when calculated over aerial (Figure 10) or total biomass (data not presented) indicates that these WUE differences are not entirely attributable to vegetative vs. reproductive partitioning. Given previous reports of wild-domestic differences in stomatal conductance and photosynthesis (Evans and Dunstone, 1970; Matesanz and Milla, 2018), this underlines the possibility of differential water-use regulation in the genus *Cicer* that warrant further investigation.

## Conclusion

This study has demonstrated large wild-domestic differences in vigor, vegetative and reproductive investment, water extraction, and WUE in the genus *Cicer* indicative of evolution under contrasting selection pressures. Dry matter allocation in wild *Cicer* is more vegetative than in domestic, which appears to be responsible for greater water extraction under terminal drought, and also greater water-use when it is freely available, but leads to a lower reproductive capacity and efficiency. While increased water extraction may be useful for chickpea improvement in water limiting environments, the wild trait combination should be disassembled as much as possible to evaluate its potential independently. To this end, wild × domestic populations have already been developed. It will be fascinating to see to what extent it is possible to recombine wild and domestic trait assemblages, whether water extraction capacity can be evaluated without simultaneously introducing low harvest index, or whether this returns a similar wild-domestic cline as observed in wild emmer x durum populations (Peleg et al., 2009).

## Data Availability Statement

The raw data supporting the conclusions of this article will be made available by the authors, without undue reservation.

## Author Contributions

JB conceived the research, helped to run the experiments, analyzed the data, and wrote the manuscript. RP, CL, SP, FB, and KW implemented the experimental program, discussed the results, and provided feedback on the manuscript. All authors contributed to the article and approved the submitted version.

### Conflict of Interest

The authors declare that the research was conducted in the absence of any commercial or financial relationships that could be construed as a potential conflict of interest.
